# Supplementary material for: New Insights into the Mechanism of Trichoderma virens-Induced Developmental Effects on Agrostis stolonifera Disease Resistance against Dollar Spot Infection
Source: J Fungi (Basel). 2022 Nov 10;8(11):1186. doi: 10.3390/jof8111186 (PMC9694513; doi:10.3390/jof8111186)
Supplement: Supplementary file 1 [file jof-08-01186-s001.zip › jof-1911343-supplementary.pdf]

Supplementary Materials

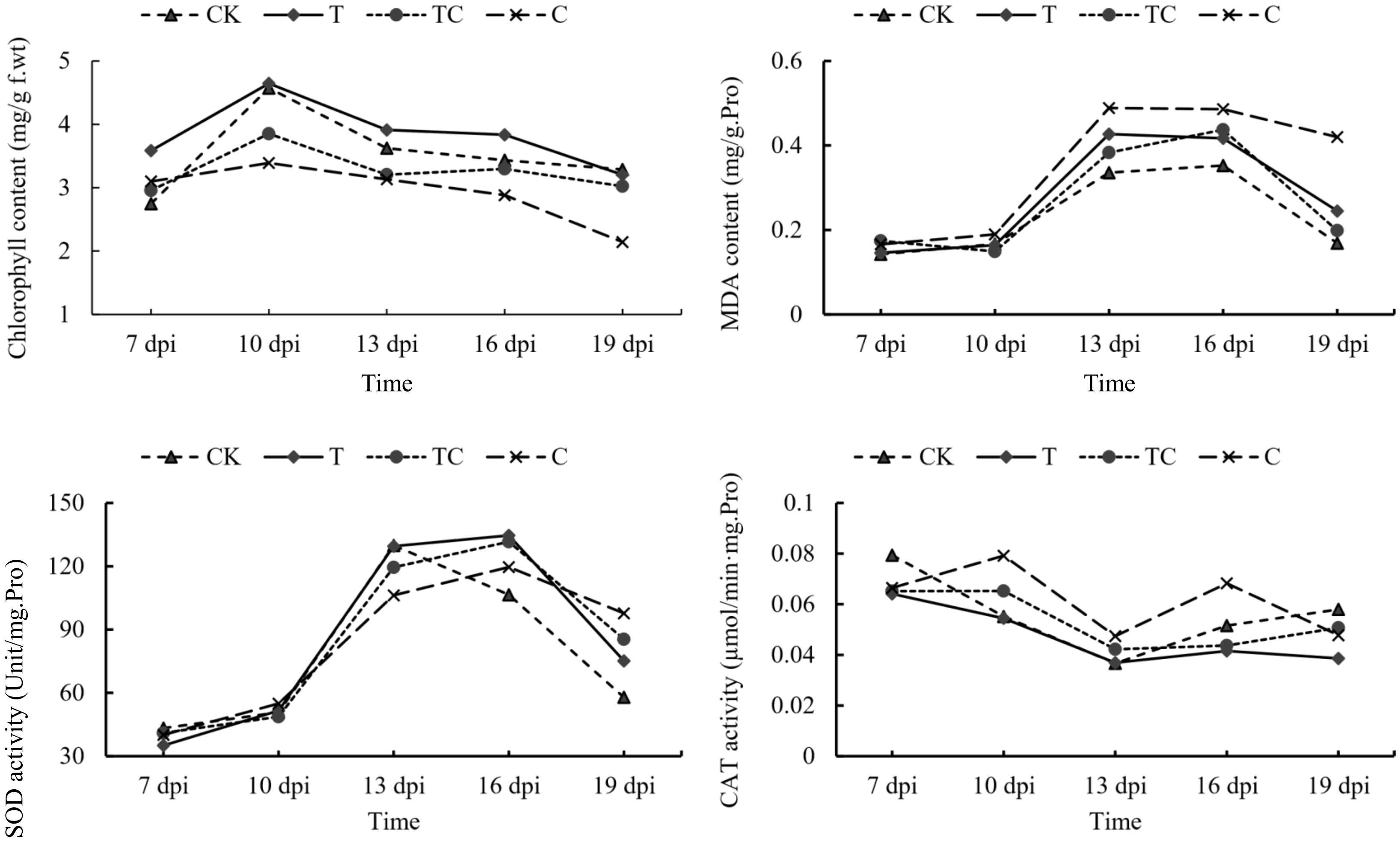

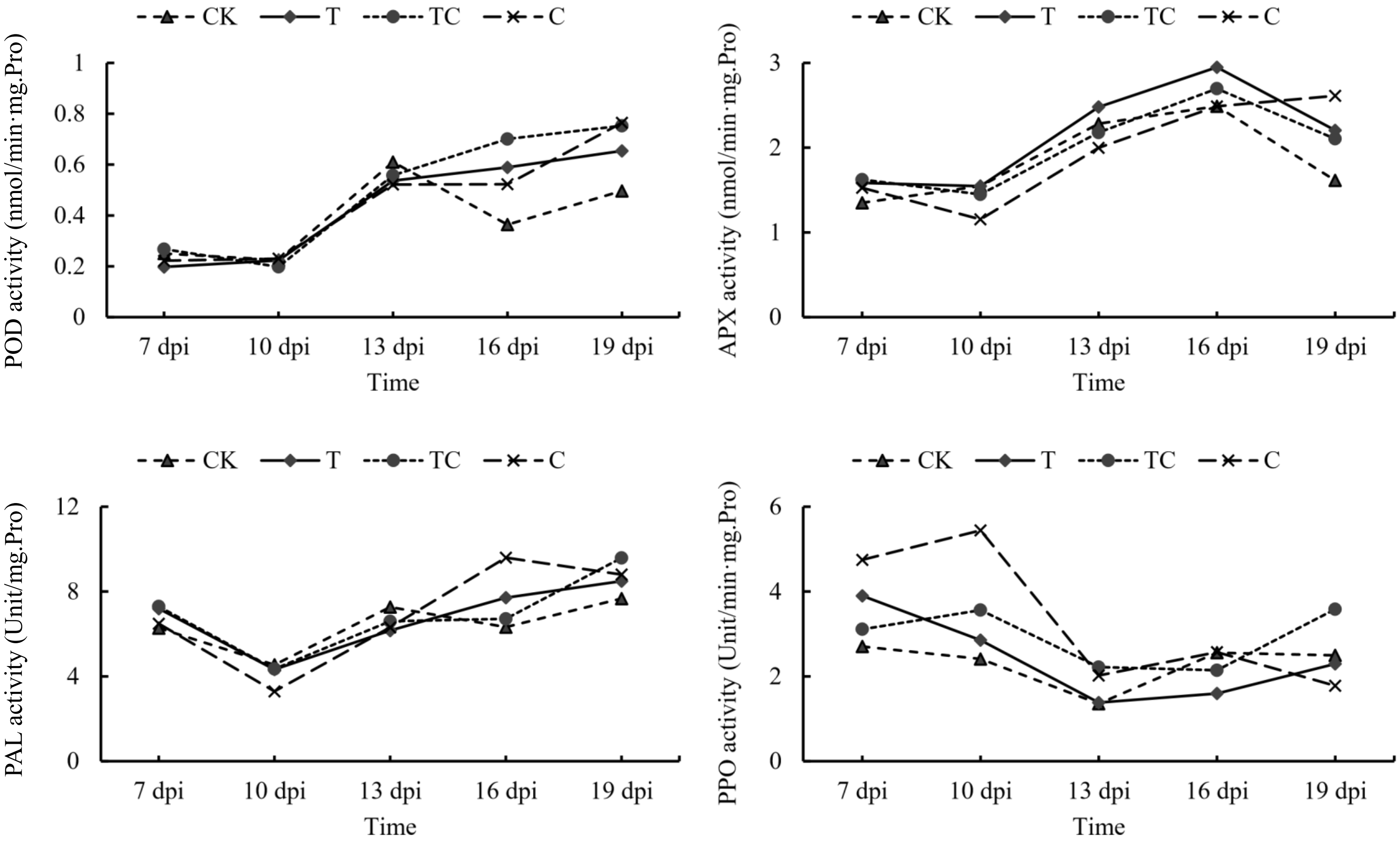

Figure S1 Physiological performance of creeping bentgrass after *T. virens* treatment and/or inoculation of *C. homoeocarpa* at 7, 10, 13, 16, and 19 dpi.

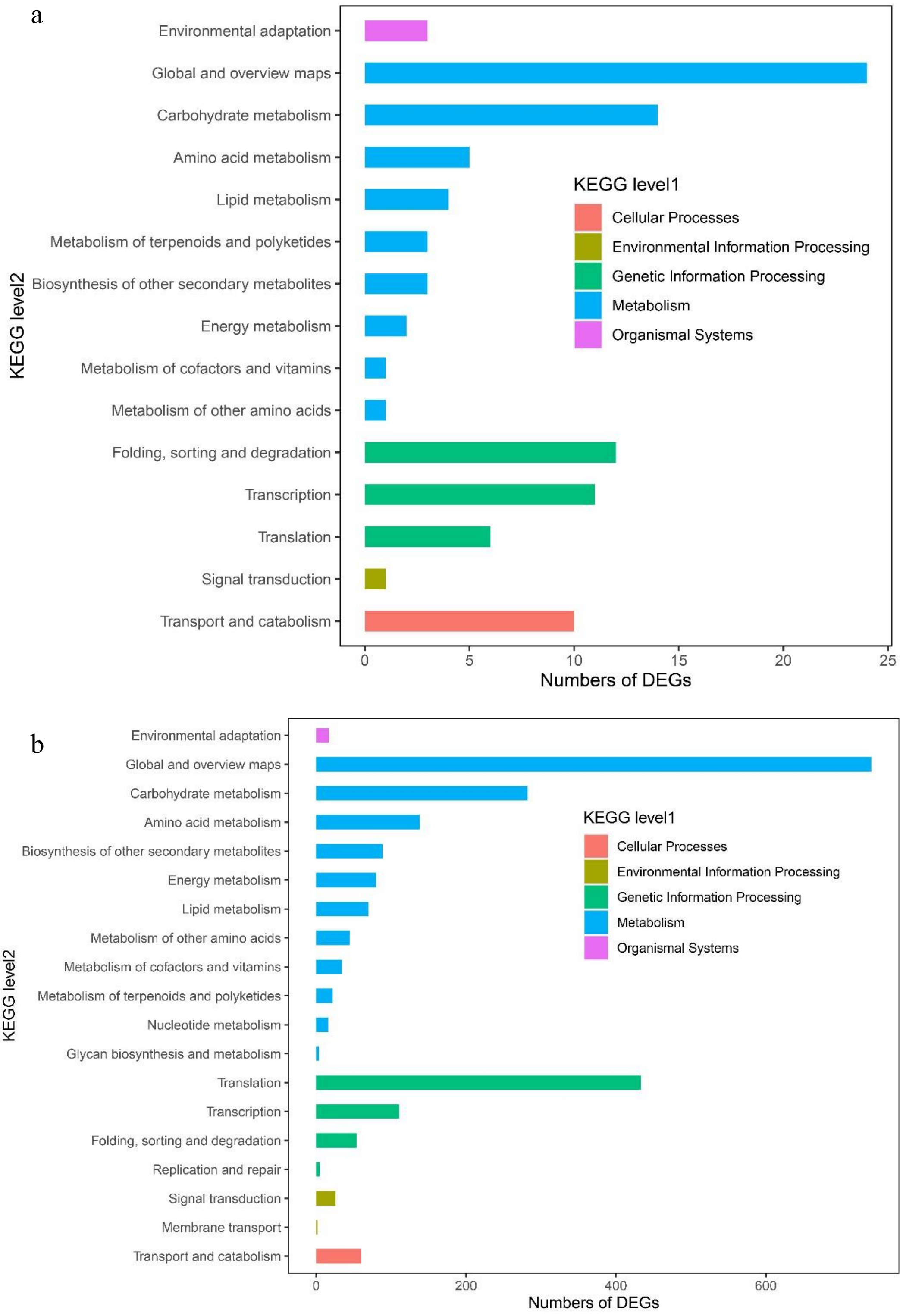

Figure S2. **The barchart of DEGs kegg class.** (a) The classification of 36 DEGs (filtered with |log2FC|>=2 & Padj<0.01) with kegg class between T. virens colonization (Tv) and control (CK). (b) The classification of 863 DEGs (filtered with |log2FC|>=2 & Padj<0.01) with kegg class between inoculation with *T. virens* and *C.homoecarp*a (Tv+Ch) and C. homoeocarpa-only inoculation (Ch).

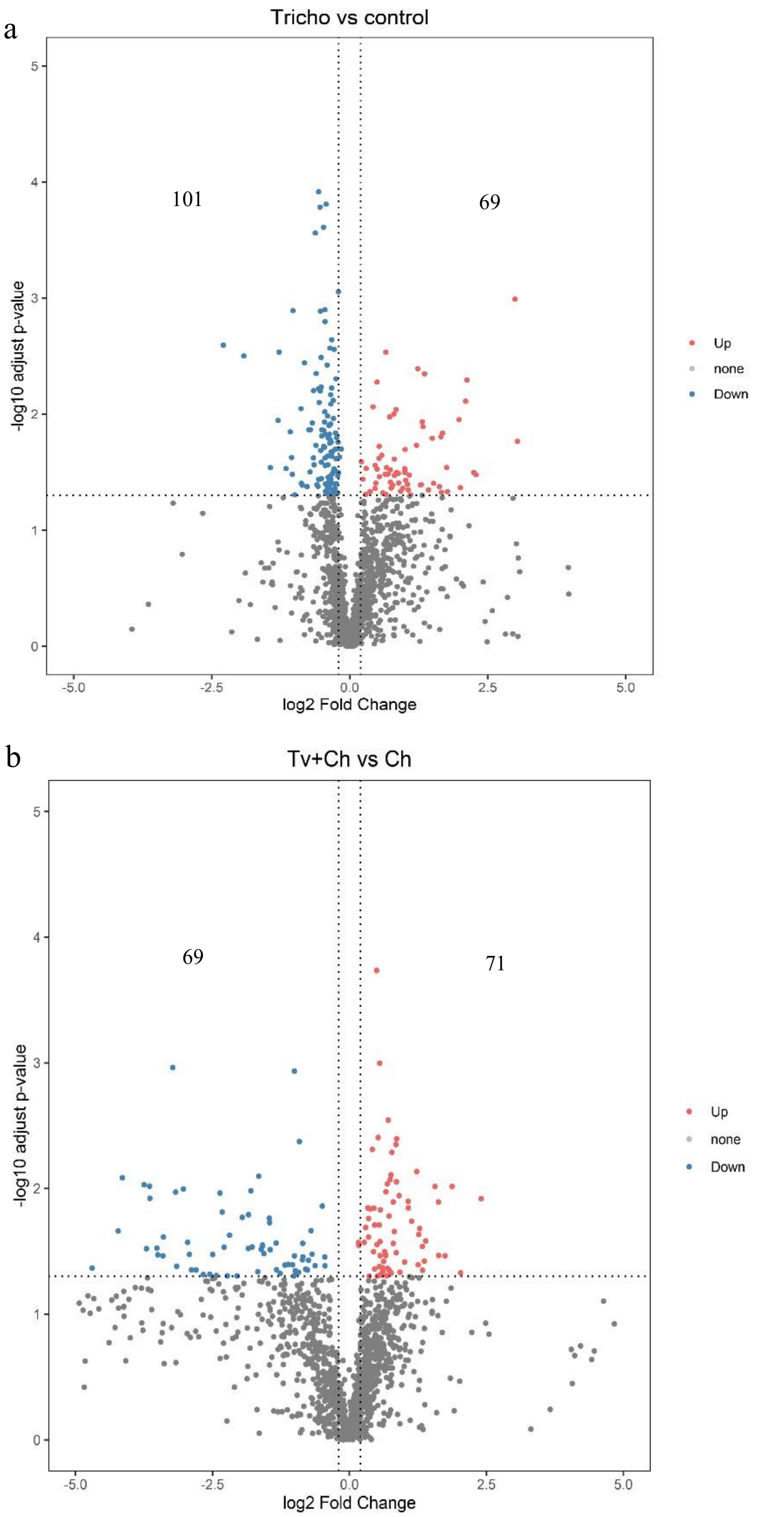

Figure S3. The analysis of metabolites with different level. (a) Volcano plot of the comparison across pre-contact with *T. virens* (Tv) and control plants (CK); (b) Volcano plot of the comparisons across inoculation with *T. virens* and *C.homoecarp*a (Tv+Ch) and C. homoeocarpa-only inoculation (Ch).

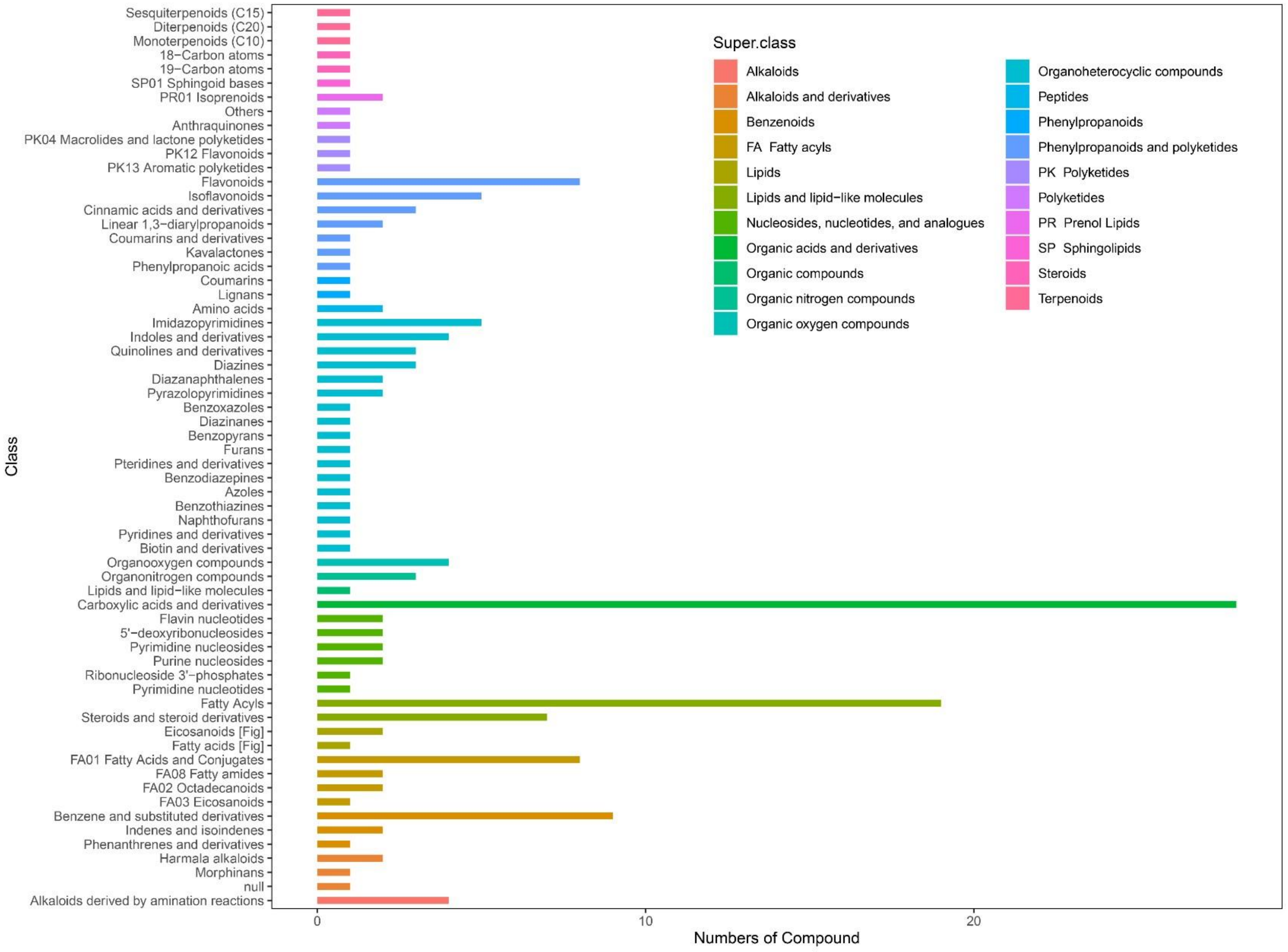

Figure S4. Barchart of class and superclass of total 177 metabolites obtained from *T. virens* 192-45.

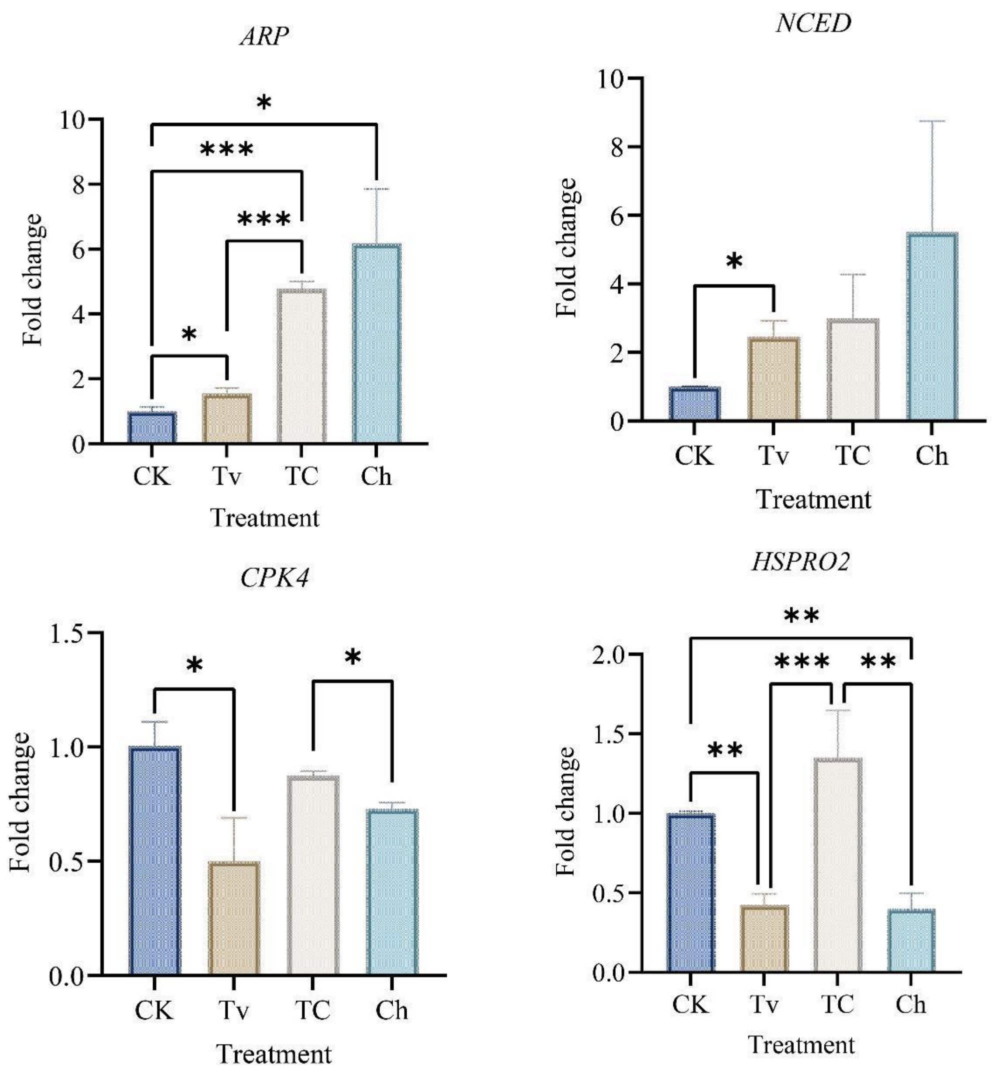

Figure S5. The expression of four candidate genes (ARP, NCED, CPK4, HSPRO2) performed by qRT-PCR.

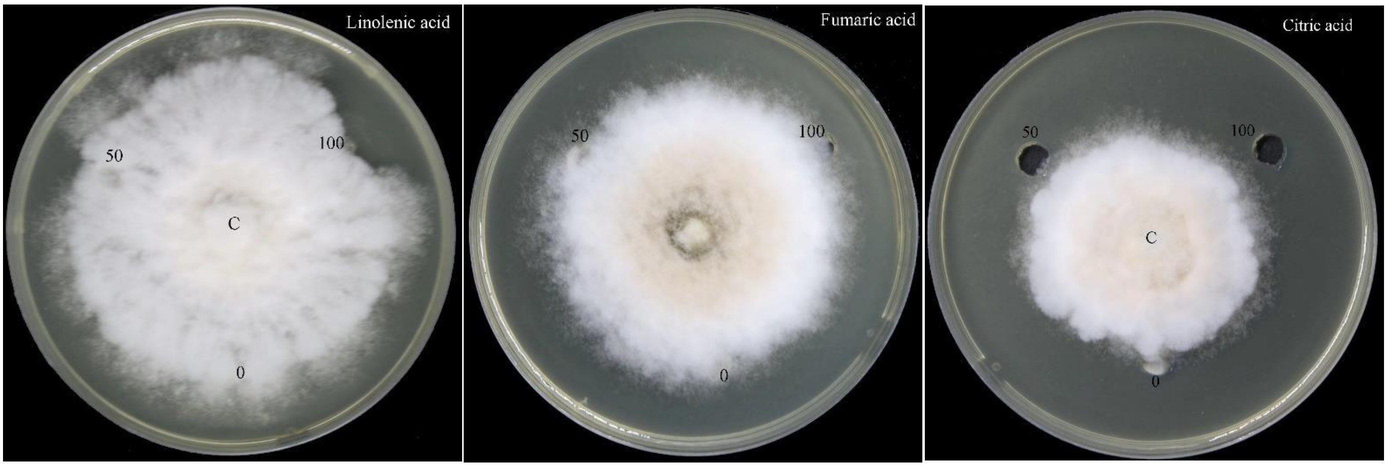

Figure S6. The disease control efficacy of linolenic, fumaric, and citric acid of 0, 50, 100 mg/mL on the growth of C. homoeocarpa
